# The effect of nitrogen management on seed yield and quality in traditional and canola-quality white mustard

**DOI:** 10.1038/s41598-024-76582-9

**Published:** 2024-10-30

**Authors:** Krzysztof Józef Jankowski, Artur Szatkowski, Dariusz Załuski

**Affiliations:** 1 Department of Agrotechnology and Agribusiness, Faculty of Agriculture and Forestry, University of Warmia and Mazury in Olsztyn, Oczapowskiego 8, 10–719 Olsztyn, Poland; 2 Department of Genetics, Plant Breeding and Bioresource Engineering, Faculty of Agriculture and Forestry, University of Warmia and Mazury in Olsztyn, Pl. Łódzki 3, 10–724 Olsztyn, Poland

**Keywords:** Yield, Fat and protein, Crude, Glucosinolates, Tocopherols, Fatty acid composition, Plant development, Plant breeding

## Abstract

The article presents the results of a three-year field study that was conducted in Poland to evaluate the yield and quality of seeds and oil from traditional (SAM) and canola-quality white mustard (SAC) in response to different N fertilizer rates (0, 40, 80, 120, and 160 kg ha^–1^). Seed yields were 25% higher in SAM than SAC. The seeds of SAC contained more crude fat (by 3%) and crude fiber (by 6%) than the seeds of SAM. In turn, the seeds of SAM were a richer source of total protein (by 7%). The content of glucosinolates (GSLs) was 8–10 times lower in the seeds of SAC than SAM. The seeds of both mustard cultivars were most abundant in γ-tocopherol (γ-T) (90–94%). The seeds of SAC were characterized by a higher content of γ-T and a lower α-T/γ-T ratio than SAM seeds. White mustard oil contained mostly MUFAs (69–75%). However, C22:1 accounted for more than 50% of MUFAs in the oil SAM. In the oil SAC, the proportion of C22:1 did not exceed 6%, whereas C18:1 accounted for nearly 85% of total MUFAs. Nitrogen fertilization induced a significant increase in seed yields (by 33%), a decrease in crude fat content (by 3–4%), and an increase in total protein content (by 4%), and crude fiber content (by 7%). Nitrogen decreased GSL levels by 31% in SAM seeds. In SAC, N fertilization induced differences in the qualitative composition of GSLs, but did not affect the total GSL content of seeds. Higher N rates increased the content of α-T and γ-T, the α-T/γ-T ratio, and total T content. Nitrogen fertilization decreased the content of C18:3 in the seeds of the SAM. In SAC, the application of N decreased the content of C18, C18:1, and C18:3, and increased the biosynthesis of C18:2, C20:1, and C22:1.

**Supplementary Information:**

The online version contains supplementary material available at 10.1038/s41598-024-76582-9.

## Introduction

The global population is expected to reach around 10 billion in 2050, and food production will have to increase by 100–110% to cater to the needs of a growing population and dietary changes resulting from economic progress^1^. According to Niggli et al.^2^, the energy (calories) derived from food crops will have to increase by 50–60%. These forecasts do not account for unsustainable consumption patterns, food waste, or increased use of agricultural biomass for non-food purposes (animal feed and biofuels)^2^. The projected increase in demand for food crops will be difficult to reconcile with the protection of non-agricultural land (forests, wetlands, deserts, mountain regions, coastal regions) and important ecosystem services (CO_2_ sequestration and protection of biodiversity). The resulting challenges are further exacerbated by climate change which, despite efforts to reduce anthropogenic greenhouse gas (GHG) emissions, poses a growing threat to biodiversity and agricultural production^3^. More than half of dietary energy comes from three cereal species (rice, maize, and wheat), and 15% of that energy is supplied by oilseed and protein crops^4,5^. Economic growth decreases the share of cereals in the human diet (in terms of metabolizable energy), while increasing the share of oilseed, protein, and sugar crops^5,6^. As a result of these changes, grain, oilseed and protein crops occupy around 87% of the world’s total arable land^7^.

African oil palm (*Elaeis guineensis* Jacq.), soybeans [*Glycine max* (L.) Merr.], rapeseed (*Brassica napus* L.), and sunflowers (*Helianthus annuus* L.) are the main sources of edible oil around the world. In the 2022/2023 growing season, these sources were responsible for around 74% of the global production of vegetable oil^5^. The cultivation of oilseed crops is centered in selected regions of the world. Nearly 90% of oil palm trees are grown in Malaysia and Indonesia. The USA, Brazil, and Argentina generate more than 80% of the global soybean supply, whereas nearly 90% of rapeseed is produced in the EU, China, Canada, and India^5^. Many regions of Europe and North America do not have supporting climates for the production of oil palm, soybeans, and sunflowers. In these regions, plants of the family *Brassicaceae* (mainly rapeseed) are the main source of edible oil^5^. However, the geographic expansion and global production of rapeseed are limited by: (i) insect damage; (ii) low competitiveness against segetal vegetation; (iii) high soil requirements; (iv) high sensitivity to abiotic stress (nutrient depletion, low temperatures during winter dormancy, high temperatures during generative development); and (v) premature silique dehiscence and seed shedding^8^. These problems can be addressed through increased use of agrochemicals, which leads to higher costs in agricultural production. In Poland, the costs associated with the production of 1 Mg of seeds of winter oilseed rape are twice higher relative to winter wheat grain^9^.

The cultivation of white mustard (*Sinapis alba* L.), also known as yellow mustard, is characterized by far fewer agronomic constraints^10^. *Sinapis alba* is a spring oilseed crop of the family *Brassicaceae*. This crop species was probably introduced to North-Western Europe, Russia, Japan, North and South America, Oceania, the Middle East, India, South Africa, and China^11,12^ from the Middle East and the Mediterranean Region^13^. In Europe, *S. alba* is grown mainly in Germany, the United Kingdom, the Netherlands, Poland, and Russia^14,15^. White mustard has many advantages over other oilseed crops. In comparison with rapeseed, white mustard is characterized by: (i) greater tolerance to drought, humidity, high and low temperatures, (ii) greater tolerance to pests and pathogens, (iii) higher competitiveness against weeds, (iv) lower soil and fertilizer requirements, (v) non-shattering siliques, and (vi) a short growing season (70–125 days)^14,16–20^.

Traditional cultivars of *S. alba* (SAM) are most widely grown. These cultivars contain 224–389 g of crude fat kg^–1^ dry matter (DM), mostly erucic acid (C22:1, 28–53%)^113,14,18,21^. Crops containing C22:1 cannot be processed into foods because this acid exerts a negative effect on the cardiovascular system, the activity of liver enzymes, and fertility^22,23^. The content of health-promoting fatty acids (FAs), including oleic acid (C18:1), linoleic acid (C18:2), and linolenic acid (C18:3), in oil derived from SAM seeds has been estimated at only 40% (12–20%, 9–12%, and 9%, respectively)^11,16,24^. Due to its long carbon chain, C22:1 is resistant to high temperature and is not solidified at low temperatures. As a result, C22:1 is a fully biodegradable, high-quality resource for the production of lubricants^25^. Erucic acid has numerous industrial applications, including in biodiesel production^16,19,26–28^. White mustard oil is characterized by high oxidative stability due to a high content of phytosterols (mainly campesterol and -sitosterol) and tocopherols (mainly γ-tocopherol)^21,29^. These compounds can reduce oxidative stress and decrease the risk of lifestyle diseases (cancer, cardiovascular and neurodegenerative diseases)^30–32^. The content of total tocopherols (Ts) in *S. alba* oil has been determined at 842–952 mg kg^–1^, including γ-T (89–91%), α-T (6–7%), and δ-T (3%). White mustard oil is free of *β*-T, and it has a very low α-T/γ-T ratio (0.07–0.08)^29,33^, which implies that it can effectively inhibit autoxidation in emulsions^34^. In the group of *Brassica* oilseed crops (BOCs), only camelina and oilseed radish oils are characterized by lower values of the α-T/γ-T ratio (0.01–0.02). It should be noted that young *S. alba* plants have considerable antioxidant potential, similarly to popular leafy vegetables such as celery (*Apium graveolens* L.), spinach (*Spinacia oleracea* L.), and Brussel sprouts [(*Brassica oleracea* L. var. *gemmifera* (DC.)]^35^.

Fat-free seed residues of SAM contain glucosinolates (GSLs). The content of GSLs ranges from 93 to 170 to even 308 µmol g^–1^ DM seeds, and sinalbin is the predominant GSL (96–98%)^36–38^. Due to high GSL content, fat-free seed residues (meal and oil cake) are not recommended in ruminant nutrition and are completely unsuitable for monogastric animals^39–42^. However, GSLs have strong biofumigant properties, which implies that fat-free seed residues of SAM can be used in the production of biopesticides^43^.

At present, SAM is cultivated mainly as a spice for the production of mustard, herbal pepper, mayonnaise, and salad dressing^40^. In SAM, the seed coat is covered with dry mucilage (crude mucilage accounts for 5% of total seed weight)^44^ which has better emulsifying properties than citrus pectin and gum Arabic^45,46^. For this reason, SAM seeds are widely used as thickeners and protein fillers in meat processing^26^. The medicinal properties of SAM have been discovered earlier than its culinary applications^40^. Ground SAM seeds combined with black mustard (*Brassica nigra* L.) and Indian mustard [*Brassica juncea* (L.) Czern.] seeds are used as herbal remedies for constipation and indigestion^14^. Essential oils and GSL extracts have warming, diuretic, expectorant, and anti-inflammatory properties; they stimulate bile production, and decrease the risk of cancer and depression^14,47–49^.

The seeds of plants of the family *Brassicaceae* are a rich source of crude fat, total protein, and biologically active substances, which is why the first breeding efforts were initiated in the 1960s to decrease the content of C22:1 in oil and GSL concentrations in seeds, thus increasing their suitability for food and feed production^50^. The first commercial cultivars of rapeseed and agrimony (*Brassica rapa* L.) with a low content of C22:1 and GSLs (double-low cultivars) were introduced in Canada in 1974 and 1977, respectively^51–53^. In 1978, the name ‘canola’ was trademarked to represent these new double-low cultivars^54^. The first commercial cultivars of canola-quality *B. juncea* were introduced in Canada in 2002 and in Australia in 2007^54,55^. In turn, the first cultivar of canola-quality *S. alba* (SAC) was developed in Poland and released for commercial cultivation only in 2012 (open-pollinated cv. Warta)^17,29,56^. Oil extracted from SAC seeds contains around 62–68% of C18:1, 12–15% of C18:2, and 11–14% of C18:3. It is more abundant in nutritionally desirable ω-3 FAs and has a more favorable ω-3/ω-6 ratio (1:1) than rapeseed oil^56^, which enhances its nutritional value.

The yields and the nutritional value of crops are influenced by plant breeding as well as soil management and agronomic treatments, mainly fertilization^57^. Fertilization not only increases yields, but also affects the nutritional value of crops. A meta-analysis conducted by Ishfaq et al.^57^ demonstrated that fertilization increased crop yields by 31% and the nutritional value of crops (content of carbohydrates, protein, fat, vitamin C, and minerals) by 12%. In BOCs, around 50–80 kg N is required to produce 1 Mg of seeds and the corresponding amount of straw^58,59^. The optimal rate of N fertilizer is highly influenced by weather conditions and the yield potential of a given species or cultivar^17,60^. According to Gan et al.^60^, traditional *S. alba* is characterized by relatively low nitrogen fertilizer use efficiency (NFUE) of 18.8 kg seeds kg^–1^ N. The NFUE in canola-quality cultivars of Indian mustard, agrimony, and rapeseed was 16%, 7%, and 40% higher, respectively. Cultivars that effectively utilize N can be incorporated into the production technology to increase yields and minimize the negative environmental impact of fertilization^61^. However, the responses of SAM and SAC to N fertilization have not been examined to date. These cultivars differ in their ability to biosynthesize the key nutrients and biologically active compounds, which could suggest that N fertilization regimes should be adapted to their individual requirements^17^. Gan et al.^60^ found that canola-quality and traditional cultivars of *B. juncea* responded differently to N fertilization. Previous research into BOCs demonstrated that agronomic practices have to be modified to reduce the concentrations of biologically active compounds with anti-nutritional properties (C22:1 and GSLs). Canola-quality cultivars of *S. alba* have been commercialized relatively recently, and little is known about their responses to agronomic factors. This is the first study to extensively analyze the responses of canola-quality *S. alba* to N fertilization (including agronomic factors and the biochemical properties of seeds and oil). The present study was undertaken to fill in the gap in knowledge about the agronomic requirements of canola-quality *S. alba* and, consequently, to improve its production technology. The study will also provide valuable information about the effect of N fertilization on the growth, development, yield, and processing suitability of *S. alba* seeds with a low content of C22:1 and GSLs. This study can also fill research gaps concerning the effectiveness of N fertilization in the production of *S. alba* with a reduced content of C22:1 and GSLs. This is an important consideration because optimal N rates will not only enable breeders to maximize yields and decrease fertilization costs, but they will also exert positive effects on (i) crop quality and (ii) the natural environment, (iii) and will promote plant adaptation to a changing climate. Therefore, the results of the present study can contribute to sustainable production of canola-quality *S. alba*.

The aim of this study was to determine the effect of N fertilization (0, 40, 80, 120, and 160 kg ha^–1^) on the growth and development (plant height, shoot diameter at the base, primary branches plant^–1^), yield components (plants m^–2^, siliques plant^–1^, seeds silique ^–1^, seed yield, straw yield, harvest index), and processing suitability (crude fat, total protein, and crude fiber content, FA profile, GSL content, and T content) of traditional *S. alba* (cv. Palma) and canola-quality *S. alba* (cv. Warta) grown in north-eastern Poland. The NFUE of both mustard cultivars was also evaluated.

## Materials and methods

### Field experiment

A small-area field experiment involving two *S. alba* cultivars was conducted in the Agricultural Experiment Station (AES) in Bałcyny (53°35’46.4’’ N, 19°51’19.5’’ E, NE Poland) in 2020–2022. The experiment had a split-plot design with two factors and three replications. The first factor was the cultivar (Palma: traditional white mustard with a high content of C22:1 and GSLs and Warta: canola-quality white mustard with a low content of C22:1 and GSLs) and the second factor was N rate (0, 40, 80, 120, 160 kg ha^–1^ in ammonium nitrate, 34% N). Nitrogen rates of ≤80 kg ha^–1^ were applied directly before sowing. Nitrogen rates of > 80 kg N ha^–1^ were split into two applications: 80 kg N ha^–1^ directly before sowing and the remainder (40 and 80 kg N ha^–1^) at the beginning of inflorescence emergence (BBCH 50, Biologische Bundesanstalt, Bundessortenamt und Chemische Industrie^62^).

Plot size was 15 m^2^ (10 by 1.5 m). The preceding crop was spring wheat (*Triticum aestivum* L.). The soil was skimmed, plowed in fall, and loosened with a cultivation unit before sowing. White mustard was sown in late March (24 March 2020, 29 March 2021, and 31 March 2022) with a plot seed drill Promar SPZ-1.5 (PW Promar sp. z o. o, Poznań, Poland) at a density of 120 pure live seeds m^–2^, with a row spacing of 19 cm, to a depth of 2–3 cm. Mineral fertilizers were applied directly before sowing: N was applied at the rates specified in the experimental design; P (enriched superphosphate, 40% P_2_O_5_) was applied at 60 kg P_2_O_5_ ha^–1^; K (potassium sulfate, 50% K_2_O, and potash salt, 60% K_2_O) was applied at 120 kg K_2_O ha^–1^; S (potassium sulfate, 18% S) was applied at 25 kg S ha^–1^. Segetal vegetation was controlled chemically at the four leaves unfolded stage (BBCH 14) with clopyralid at 43.5 g ha^–1^ and picloram at 23.5 g ha^–1^. Insecticides were applied four times during the growing season: (i) lambda-cyhalothrin at 5 g ha^–1^ (BBCH 14); (ii) acetamiprid at 20 g ha^–1^ + lambda-cyhalothrin at 6 g ha^–1^ (BBCH 50); (iii) thiacloprid at 60 g ha^–1^ + deltamethrin at 6 g ha^–1^ (BBCH 55); and (iv) cypermethrin at 25 g ha^–1^ (BBCH 65). Fungicides were not applied due to the low prevalence of pathogens. White mustard plants were harvested at the beginning of August (4 August 2020, 6 August 2021, 10 August 2022) with a small-plot harvester Wintersteiger Classic 1540–447 (Wintersteiger Holding AG, Ried im Innkreis, Austria).

The experiment was conducted on Haplic Luvisol^63^ formed from boulder clay. The chemical composition of soil was determined in samples collected at a depth of 0–20 cm (three samples per plot). White mustard was grown on slightly acidic soil (pH 5.8–6.4). The content of C_org_ and N_t_ was determined in the range of 10–13 and 1.08–1.24 g kg^–1^, respectively. The content of plant-available nutrients in the arable layer was determined at: 56.6–96.8 mg P kg^–1^, 137.0–149.4 mg K kg^–1^, 39.0–55.0 mg Mg kg^–1^, and 4.0–8.2 mg $\:{\text{S}\text{O}}_{4}^{2-}$kg^–1^. Soil pH was measured with a digital pH meter with temperature compensation (20 °C) in deionized water and 1 M KCl dm^–3^ (5:1). The content of C_org_ in soil was determined by the modified Kurmies method (UV-1201 V spectrophotometer, Shimadzu Corporation, Kyoto, Japan)^64^. The content of N_t_ was determined with the Kjeldahl distillation method with a KjelFlex K-360 analyzer (Büchi Labortechnik AG, Flawil, Switzerland). Plant-available P and K were extracted with calcium lactate (Egner-Riehm method) by vanadium-molybdenum yellow spectrophotometry (UV-1201 V spectrophotometer, Shimadzu Corporation, Kyoto, Japan) and atomic emission spectrometry (Flame Photometers, BWB Technologies Ltd., Newbury, UK), respectively^64^. Magnesium was extracted with 0.01 mol of calcium chloride and quantified by atomic absorption spectrophotometry (AAS1N, Carl Zeiss, Jena, Germany)^64^. The content of sulfide sulfur was by determined by nephelometry^65^ after extraction in an acetate buffer (UV-1201 V spectrophotometer, Shimadzu Corporation, Kyoto, Japan). The chemical properties of soil were determined in the laboratory of the District Chemical-Agricultural Station in Olsztyn.

### Plant materials

Plant height, shoot diameter at the base, primary branches plant^–1^, plants m^–2^, siliques plant^–1^, and seeds silique^–1^ were determined in early stages of ripening (BBCH 80–82). The number of plants m^–2^ was determined by counting plants in two middle rows of each plot (5 measurements along a 1 m section). Plant height, shoot diameter at the base, primary branches plant^–1^ and siliques plant^–1^ were determined in 10 plants sampled from each plot (two middle rows). The number of seeds silique^–1^ was determined in 20 siliques sampled from 10 plants in two middle rows of each plot. Thousand-seed weight (TSW), seed yields, and straw yields were determined after harvest. Directly after harvest, seed and straw samples of 100 g each were collected to determine dry matter (DM) content. The samples were dried in an FD 53 oven (Binder GmbH, Tuttlingen, Germany) until constant weight. The DM content of seeds and straw was calculated according to the procedure described by Szatkowski et al.^66^. Seed and straw yields and TSW were calculated for 91% DM content. The harvest index (HI) and NFUE were calculated according to the procedures described by Rathke et al.^67^, Malhi et al.^68^, and Jankowski et al.^69^.

### Processing suitability of seeds

The content of crude fat, total protein (in g kg^− 1^ DM seeds), and GSLs (in µM g^− 1^ DM seeds) was determined in an infrared analysis with the use of a reflectance spectrophotometer NIRS Systems 6500 (FOSS Inc., Silver Spring, MD, United States) within the range of 400–2500 nm. The content of crude fiber (in g kg^− 1^ DM seeds), acid detergent fiber (ADF), and neutral detergent fiber (NDF) (in %) was determined with the Ankom 200 fiber analyzer (Ankom Technology, Macedon, NY, United States) according to official method BA 6a-05 of the American Oil Chemists Society, AOCS^70^. Fatty acid methyl esters (FAMEs) were prepared according to the procedure described by Jankowski et al.^69^. The obtained FAMEs were quantified using the Agilent 7890 A gas chromatography system (Agilent Technologies Inc., Palo Alto, CA, United States) with a FID detector and a DB-23 capillary column (30 m in length, 0.25 mm, 0.25 μm) with an operating temperature of 200 °C (injector and detector temperature was 220 °C) and hydrogen as the carrier gas.

The content of α-T, β-T, γ-T, and δ-T in seeds was determined according to Polish Standard^71^ under limited exposure to sunlight. Seed sample were prepared according to the procedure described by Gawrysiak-Witulska et al.^29^ and analyzed by reversed-phase high-performance liquid chromatography RP-HPLC (Shimadzu Corporation, Kyoto, Japan) on a short Phenomenex Nucleosil C_18_ column (250 × 4.6 mm, 5 μm). The mobile phase was composed of CH_3_OH and H_2_O (95:5 v/v) (HPLC grade Sigma-Aldrich Chemie GmbH, Munich, Germany). The flow rate was 1 cm^3^ min^–1^. The RF detector was operated at excitation and emission wavelengths of E_x_ 293 and E_m_ 326, respectively, with a 20 µL loop. The following external standards were used: (±)-α-T (DL-all-rac α-T), β-T, (+)-γ-T, (+)-δ-T (p.a. Sigma-Aldrich Chemie GmbH, Munich, Germany).

### Statistical analysis

The statistical analysis of the results was divided into two parts. The first part involved traits that were not related to the division of the analyzed cultivars of *S. alba* (traditional and canola-type) in the three-year experiment. Data were processed statistically by three-way analysis of variance (ANOVA) for a split-split plot design, where growing seasons were assigned to whole plots, cultivars were assigned to subplots, and N rates were assigned to sub-subplots (Eq. 1).

1 $$\:{y}_{ijkl}=m+{a}_{i}+{r}_{j}+{e}_{ij}^{1}+{b}_{k}+{ab}_{ik}+{e}_{ijk}^{2}+{c}_{l}+{ac}_{il}+{bc}_{kl}+{abc}_{ikl}+{e}_{ijkl}^{3}$$

where *y*_ijkl_ is the observed *i*-th level of factor A (growing season), *k*-th level of factor B (cultivar), and *l*-th level of factor C (N rate) in the *j*-th replication; *m* is the mean value; *r*_*j*_ is the replication; *ab*_*ik*_, *ac*_*il*_, *bc*_*kl*_ and *abc*_*ikl*_ are the interactions. The errors *e*^1^_*ij*_, *e*^2^_*ijk*_ and *e*^3^_*ijkl*_ are normally distributed with zero mean and σ^2^_*ij*_, σ^2^_*ijk*_ and σ^2^_*ijkl*_ variance^72^ (Table S1).

The qualitative factors that differentiated the examined *S. alba* cultivars (FA profile, quantitative and qualitative composition of GSLs) were analyzed relative to N rates, separately for each cultivar (Palma and Warta) (Table S2).

Prior to performing all of the above analyses, the data were tested for the assumption of homoscedasticity using Bartlett’s test and the data did not require any transformations before performing ANOVA. All analyses were performed in the Statistica 13.3 program^73^ at a significance level of* α* = 0.05.

## Results

### Weather conditions

**Fig. 1:**
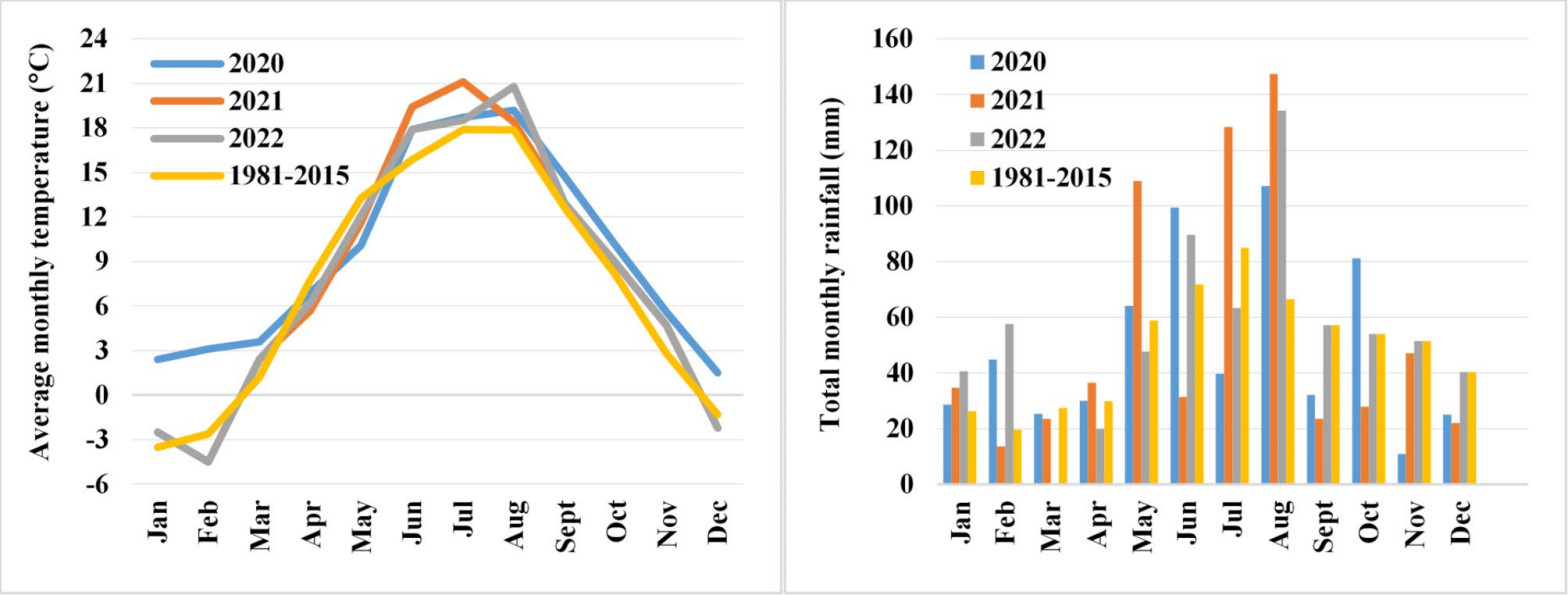
Average monthly temperature and total monthly precipitation during the growing seasons of *S. alba* (2020–2022) and the long-term average (1981–2015) determined by an automatic weather station in AES Bałcyny (PM Ecology Ltd., Gdynia, Poland).

During the field experiment, mean annual temperature exceeded the long-term average by 2.0 °C in 2020, 0.5 °C in 2021, and 0.4 °C in 2022 (Fig. 1). Lower precipitation levels were noted in years with high mean annual temperature. The lowest precipitation (approximating the long-term average of 588 mm) was observed in the first year of the experiment which was characterized by the highest mean annual temperature (9.5 °C). In years 2 and 3 (when mean annual temperatures were 1.5 and 1.6 °C lower, respectively, than in year 1), precipitation exceeded the long-term average by 10% and 12%, respectively. The growing seasons (April–August) during the three-year field experiment were also characterized by varied temperatures and precipitation levels. In the first growing season, mean daily temperature approximated the long-term average (14.5 °C). In the second and third growing season, mean daily temperatures were 0.5 °C higher than the long-term average. In all years of the study, mean daily temperatures before inflorescence emergence (April and May) were lower than the long-term average (by 0.9–2.1 °C and 1.2–3.2 °C, respectively). Mean daily temperatures after inflorescence emergence until harvest (June, July, and August) exceeded the long-term average by 2.0–3.5 °C (2020), 0.6–2.8 °C (2021), and 0.5–2.9 °C (2022).

Considerable variations in precipitation levels and distribution were also noted across growing seasons. In year 1, total precipitation between April and August approximated the long-term average (311 mm). In the second and third growing season, total precipitation exceeded the long-term average by 45% and 17%, respectively. Rainfall distribution was least favorable in year 3, when precipitation in April, May, and July was 33%, 19%, and 25% lower, respectively, than the long-term average. In the first and second year, precipitation was below the long-term average only in July and June (by 53% and 56%, respectively) (Fig. 1).

### Stand architecture

**Fig. 2:**
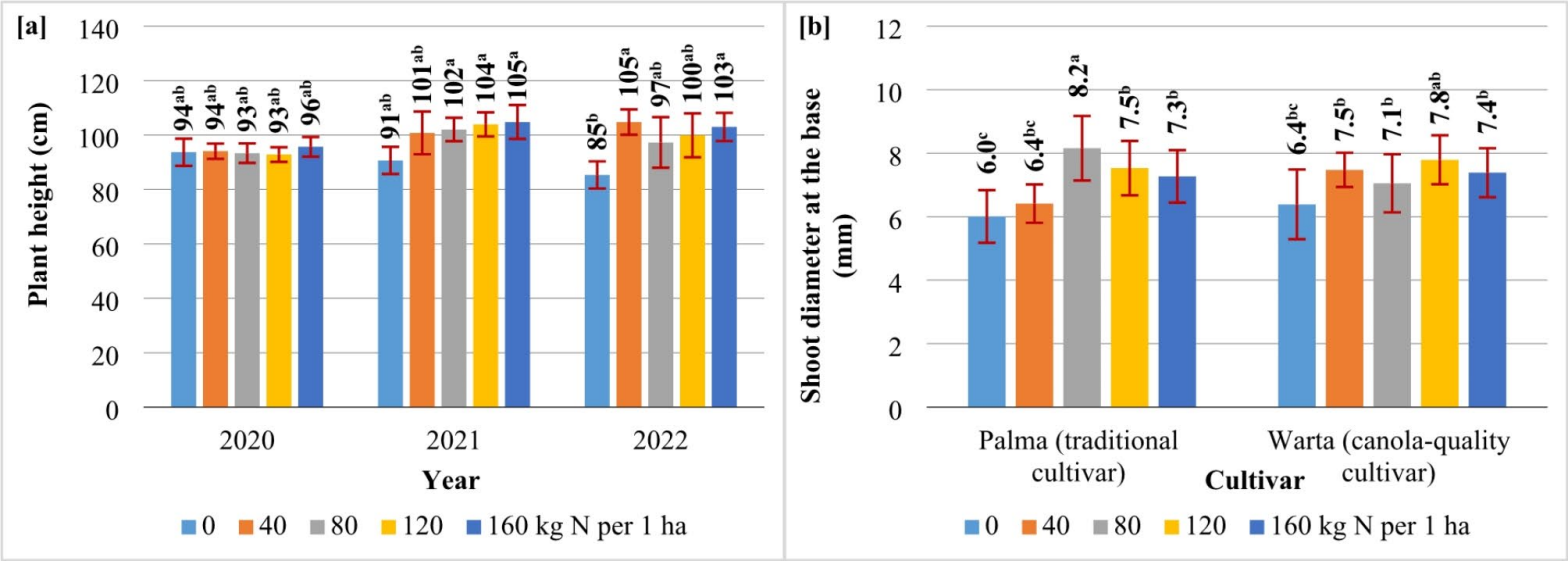
The effect of nitrogen fertilization on plant height (**a**) and shoot diameter (**b**) in *S. alba*. Means with the same letters do not differ significantly at *p* ≤ 0.05 in Tukey’s test.

Both cultivars of *S. alba* produced the longest stems (98–100 cm) with the largest shoot diameter at the base (7.9–8.0 mm) and the highest number of primary branches (4.9–6.5 primary branches plant^–1^) in the first and second year of the study. The growth habit of *S. alba* plants was least desirable in year 3 (relatively short, thin, and weakly branched stems) (Table 1) which was characterized by the least supportive rainfall distribution (Fig. 1). Cultivar Palma (SAM) produced taller plants (by 13 cm) with fewer branches (by 13%) than cv. Warta (SAC). The N rate of 40 kg ha^–1^ exerted the most stimulatory effect on plant height in both cultivars (stem length increased by 11% relative to the control treatment) (Table 1). The N rate of 40 kg N ha^–1^ induced the greatest increase in plant height (by 24%) in year 3 (Fig. 2a). Nitrogen exerted a significant influence on stem thickness only in SAM plants (shoot diameter at the base increased by 37% in response to 80 kg N ha^–1^) (Fig. 2b).

**Table 1:** Morphometric parameters of *S. alba* plants.

Specification	Plant height (cm)	Shoot diameter at the base (mm)	Primary branches plant^–1^
Years			
2020	98^b^	8.0^a^	4.9^b^
2021	100^a^	7.9^a^	6.5^a^
2022	94^ab^	5.6^b^	3.7^c^
Cultivar, mean for 2020–2022			
Palma (traditional cultivar)	104^a^	7.1	4.7^b^
Warta (canola-quality cultivar)	91^b^	7.2	5.4^a^
Nitrogen rate (kg ha^–1^), mean for 2020–2022			
0	90^b^	6.2^b^	4.8
40	100^a^	6.9^ab^	5.0
80	98^a^	7.6^a^	5.3
120	99^a^	7.7^a^	5.0
160	101^a^	7.3^a^	5.2

Means with the same letters do not differ significantly at *p* ≤ 0.05 in Tukey`s test.

### Yield components

Stand density at harvest was determined by cultivar. Weather conditions and N fertilization had no significant effect on stand density at harvest (Table S1). Stand density was 25% higher in cv. Palma than in cv. Warta. In turn, cv. Warta produced 28% more siliques plant^–1^ than cv. Palma. In both cultivars of *S. alba*, the number of siliques plant^–1^ continued to increase up to the highest N rate (160 kg ha^–1^) (Table 2). The number of seeds silique^–1^ was one of the most stable yield components in both cultivars. This parameter was significantly differentiated only by weather conditions (Tables S1 and 2). Both cultivars produced the largest seeds in the third year of the experiment (7.13 g), and TSW was 8% and 16% lower in years 1 and 2, respectively. During the entire experiment, TSW was 29% higher in SAM than SAC on average. Nitrogen rates of ≥80 kg ha^–1^ exerted a negative effect on TSW in both *S. alba* cultivars (decrease of 3–4%) (Table 2).

**Table 2:** Yield components in *S. alba*.

Specification	Plants m^–2^	Siliques plant^–1^	Seeds silique^–1^	TSW (g)
Years				
2020	61.9	65.7	4.9^b^	6.53^b^
2021	59.8	70.9	5.5^a^	5.96^c^
2022	61.3	69.7	5.0^b^	7.13^a^
Cultivar, mean for 2020–2022				
Palma (traditional cultivar)	67.5^a^	60.3^b^	5.1	7.38^a^
Warta (canola-quality cultivar)	53.9^b^	77.2^a^	5.1	5.70^b^
Nitrogen rate (kg ha^–1^), mean for 2020–2022				
0	57.3	59.1^b^	5.1	6.70^a^
40	62.4	63.1^b^	5.1	6.66^ab^
80	61.2	68.9^ab^	5.1	6.44^b^
120	63.3	72.4^ab^	5.2	6.47^b^
160	58.7	80.4^a^	5.2	6.43^b^

TSW − 1000-seed weight. Means with the same letters do not differ significantly at *p* ≤ 0.05 in Tukey’s test.

### Yield and the harvest index

In north-eastern Poland, the seed yields of *S. alba* ranged from 1.24 to 1.49 Mg ha^–1^. Seed yields were 25% higher (by 0.30 Mg ha^–1^) in SAM than in SAC (Table 3). Traditional white mustard was characterized by higher yield potential in all years of the experiment (Table S1). Both *S. alba* cultivars responded similarly to N fertilization (Table S1). A significant increase in seed yield (by 33% relative to the control treatment) was noted only after the application of 120 kg N ha^–1^ (Table 3). The NFUE ranged from 16.4 to 19.3 kg seeds kg^–1^ N. This parameter was 19% higher in SAM than in SAC. In both cultivars, the NFUE peaked (31.8 kg seeds kg^–1^ N) in response to 40 kg N ha^–1^. An increase in the N rate to 80 and 120–160 kg ha^–1^ decreased the NFUE by 47% and 62–70%, respectively (Table 3).

**Table 3:** Biomass yields and the harvest index in *S. alba*.

Specification	Seed yield (Mg ha^–1^)	NFUE (kg seeds per 1 kg *N*)	Straw yield (Mg ha^–1^)	Harvest index
Years				
2020	1.24	16.4	1.75^b^	0.44^b^
2021	1.30	17.1	1.37^c^	0.50^a^
2022	1.49	19.3	2.02^a^	0.44^b^
Cultivar, mean for 2020–2022				
Palma (traditional cultivar)	1.49^a^	19.1^a^	1.97^a^	0.45
Warta (canola-quality cultivar)	1.19^b^	16.1^b^	1.46^b^	0.47
Nitrogen rate (kg ha^–1^), mean for 2020–2022				
0	1.10^b^	–	1.43^b^	0.45
40	1.27^ab^	31.8^a^	1.72^ab^	0.45
80	1.34^ab^	16.8^b^	1.76^ab^	0.45
120	1.46^a^	12.2^c^	1.85^a^	0.46
160	1.54^a^	9.6^d^	1.81^a^	0.48

NFUE - nitrogen fertilizer use efficiency. Means with the same letters do not differ significantly at *p* ≤ 0.05 in Tukey’s test.

**Fig. 3:**
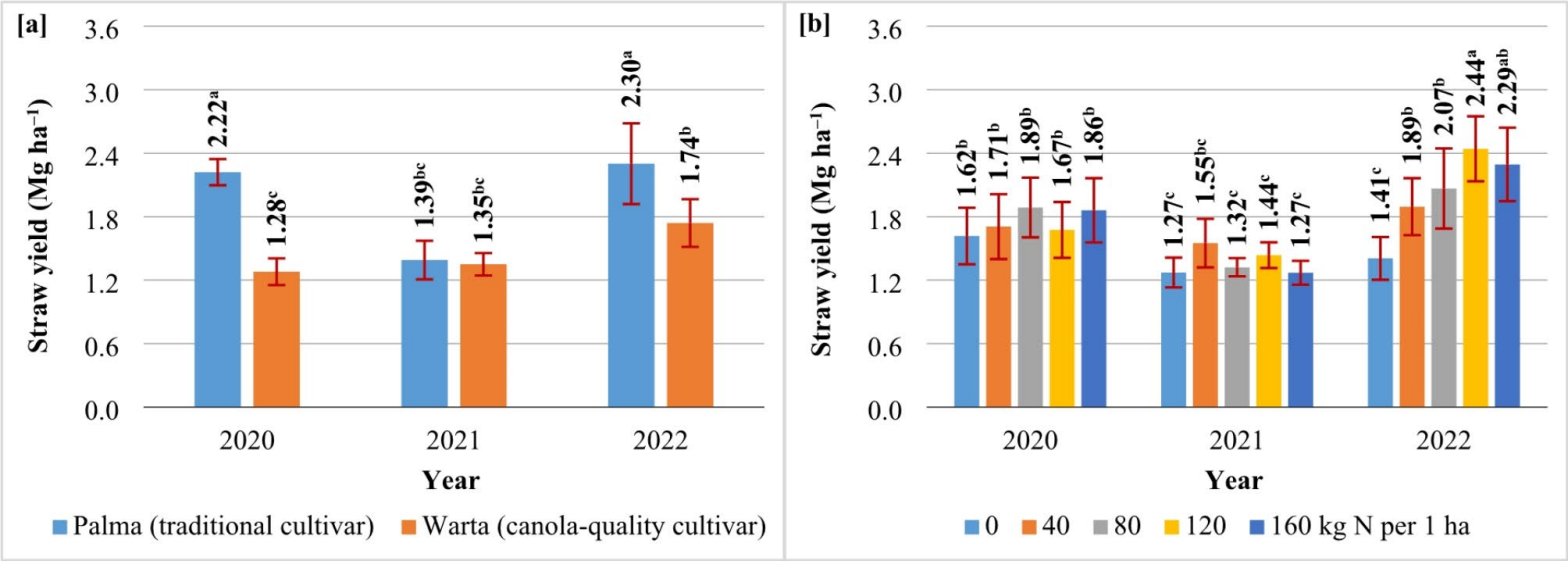
The influence of cultivar (**a**) and nitrogen fertilization (**b**) on straw yields in *S. alba* (2020, 2021, 2022). Means with the same letters do not differ significantly at *p* ≤ 0.05 in Tukey’s test.

Straw yields ranged from 1.37 to 1.75 (2020, 2021) to 2.02 Mg ha^–1^ (2022). On average, straw yields were 35% higher in SAM than in SAC (Table 2). The straw yields of the analyzed cultivars did not differ only in year 2 (Fig. 3a). The N rate of 120 kg ha^–1^ increased straw yields by 29% on average in both cultivars (Table 3), which was attributed to a very high increase (73%) in straw yields in only one growing season (2022) under the influence of 120 kg N ha^–^ (Fig. 3b).

**Fig. 4:**
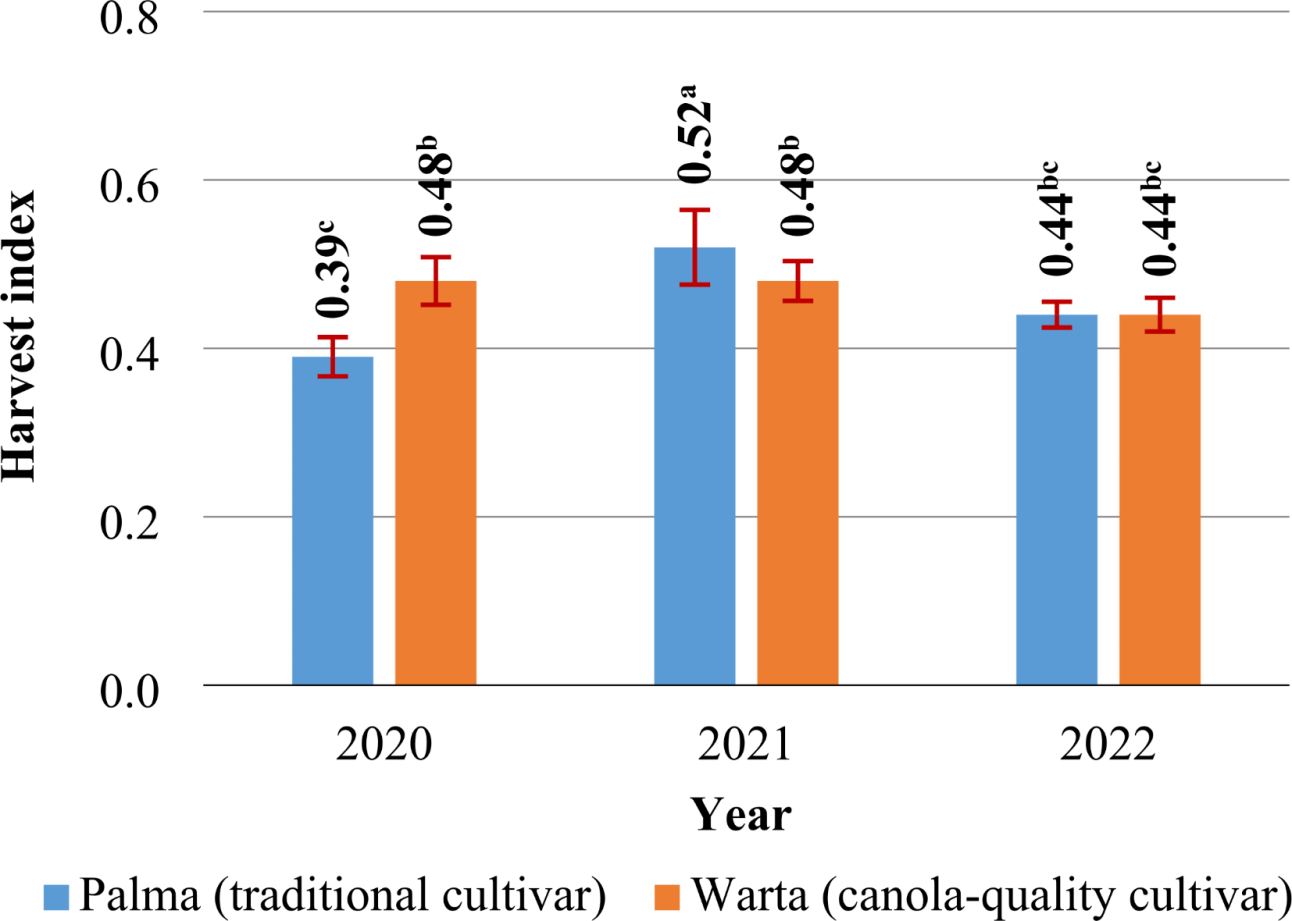
The harvest index *S. alba* cultivars (2020, 2021, 2022). Means with the same letters do not differ significantly at *p* ≤ 0.05 in Tukey’s test.

The harvest index (HI) *S. alba* ranged from 0.44 (2020, 2022) to 0.50 (2021) (Table 3). The HI of SAM and SAC differed only in years 1 and 2. In year 1, HI was 23% higher in SAC than in SAM. In year 2, the ratio of seeds to total biomass was 8% higher in the traditional cultivar than in the canola-quality cultivar of white mustard (Fig. 4).

### Seed and oil quality

#### Fat, protein, and fiber content of seeds

**Fig. 5:**
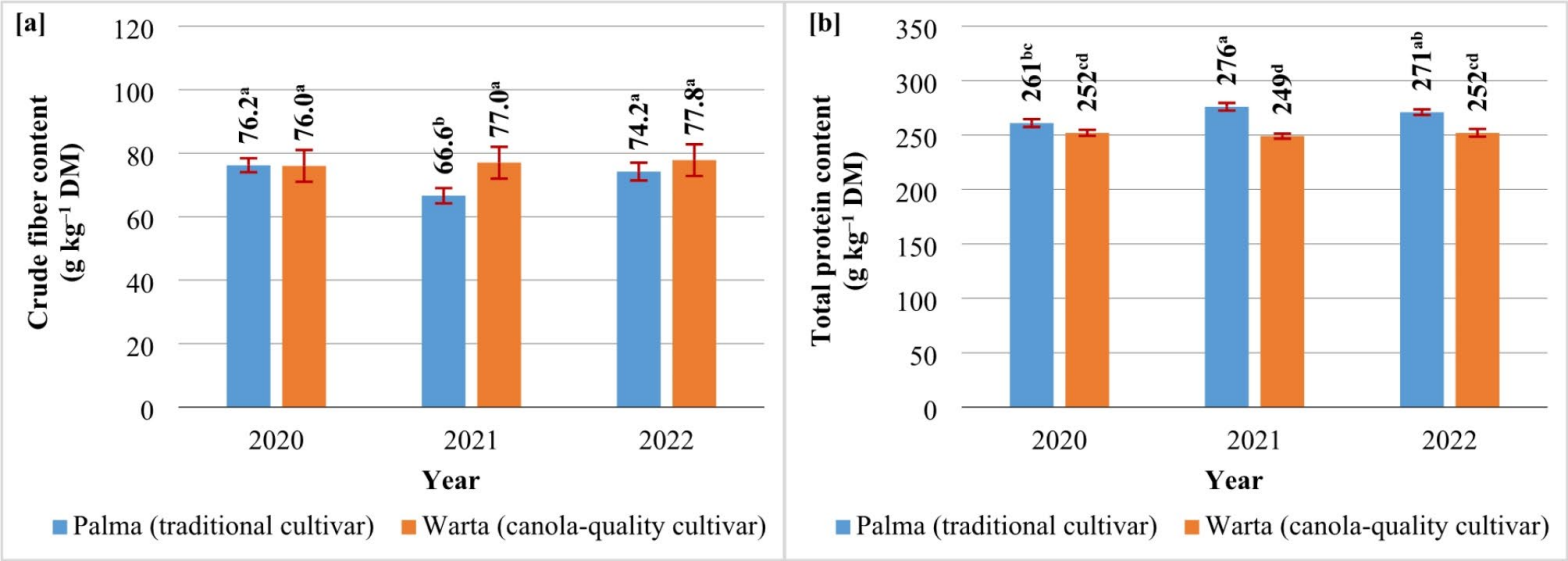
Content of crude fiber (**a**) and total protein (**b**) in the seeds of the analyzed *S. alba* cultivars (2020, 2021, 2022). Means with the same letters do not differ significantly at *p* ≤ 0.05 in Tukey’s test.

The seeds of the traditional cultivar of *S. alba* contained 303–310 g of crude fat kg^–1^ DM, 256–263 g of total protein kg^–1^ DM, and 72–76 g of crude fiber kg^–1^ DM, including 14.9–15.8% of ADF and 20.9–23.1% of NDF (Table 4). The seeds of canola-quality white mustard contained 3% more crude fat and 6% more crude fiber than the traditional cultivar. The greatest differences in the crude fiber content of SAM and SAC were observed in the second growing season (72 vs. 77 g kg^–1^ DM, respectively) (Fig. 5a). In turn, SAM seeds were a richer source of total protein (by 7%) than SAC seeds (Table 4). The most notable differences in the total protein content of SAM and SAC seeds were noted in years 2 and 3 (Fig. 5b).

**Table 4:** Content of nutrients in *S. alba* seeds.

Specification	Crude fat content (g kg^–1^ DM)	Total protein content (g kg^–1^ DM)	Crude fiber content (g kg^–1^ DM)	ADF (%)	NDF (%)
Years					
2020	310	256^b^	76.9^a^	15.8^a^	23.1^a^
2021	303	263^a^	71.8^b^	15.2^b^	21.0^b^
2022	309	261^ab^	76.0^a^	14.9^b^	20.9^b^
Cultivar, mean for 2020–2022					
Palma (traditional cultivar)	302^b^	269^a^	72.3^b^	15.1	21.3^b^
Warta (canola-quality cultivar)	312^a^	251^b^	76.9^a^	15.5	22.0^a^
Nitrogen rate (kg ha^–1^), mean for 2020–2022					
0	315^a^	255^b^	72.0^b^	14.5^b^	21.7
40	311^ab^	257^ab^	73.2^ab^	15.0^ab^	21.7
80	306^b^	260^ab^	75.1^ab^	15.2^ab^	21.8
120	303^bc^	262^ab^	77.0^a^	15.8^a^	21.7
160	301^c^	266^a^	75.6^ab^	15.9^a^	21.4

*ADF* acid detergent fiber, *NDF* eutral detergent fiber. Means with the same letters do not differ significantly at *p* ≤ 0.05 in Tukey’s test.

Nitrogen fertilization decreased crude fat content and increased total protein content in the seeds of both white mustard cultivars (Table 4). In both cultivars, the content of crude fat decreased (by 3–4%) already in response to 80 kg N ha^–1^. In turn, a significant increase in total protein content (by 4%) was observed only after the application of 160 kg N ha^–1^. In both cultivars of *S. alba*, 120 kg N ha^–1^ induced a significant increase (7%) in crude fiber content and an increase in ADF content (1.3% points, %p) (Table 4).

#### Glucosinolate content

Sinalbin was the predominant GSL in SAM seeds (141.8–203.2 µM g^–1^ DM) (Table 5). The content of aliphatic GSLs (progoitrin and napoleiferin) and indole GSLs (glucobrassicin and 4-hydroxyglucobrassicin) was determined at 3.3–7.1 and 0.4–0.8 µM g^–1^ DM, respectively. In SAM, N fertilization decreased the content of aliphatic GSLs (progoitrin and napoleiferin) and aromatic GSLs (sinalbin) in seeds. The content of progoitrin and napoleiferin decreased up to the N rate of ≥80 kg N ha^–1^ (by 49% and 47%, respectively). In turn, sinalbin content decreased up to the N rate of 160 kg N ha^–1^ (by 30%, i.e. by 61.4 µM g^–1^ DM). Total GSL content decreased by 31% (by 64.9 µM g^–1^ DM) in response to the highest N rate of 160 kg N ha^–1^ (Table 5).

**Table 5:** Content of glucosinolates in *S. alba* seeds (µM g^–1^ DM), mean for 2020–2022.

Nitrogen rate (kg ha^–1^)	GNA	PRO	NPL	GBS	4HGBS	SNB	TROP	Aliphatic GSLs	Indole GSLs	Aromatic GSLs	Total-GSLs
Cv. Palma (traditional cultivar)											
0	0.00	3.57^a^	3.57^a^	0.23	0.17^c^	203.17^a^	0.00	7.13^a^	0.40	203.17^a^	210.70^a^
40	0.00	3.10^a^	3.10^b^	0.33	0.33^b^	184.23^b^	0.00	6.20^a^	0.67	184.23^ab^	191.10^a^
80	0.00	1.74^cd^	1.70^c^	0.27	0.50^a^	165.07^c^	0.00	3.44^b^	0.77	165.07^b^	169.28^b^
120	0.00	1.80^c^	1.87^c^	0.23	0.47^a^	152.32^cd^	0.00	3.67^b^	0.70	152.32^bc^	156.69^bc^
160	0.00	1.54^d^	1.73^c^	0.27	0.51^a^	141.79^d^	0.00	3.28^b^	0.78	141.79^c^	145.84^c^
Cv. Warta (canola-quality cultivar)											
0	0.19^b^	8.37^a^	8.37^a^	2.23	2.13^b^	0.00	0.47^a^	16.92^a^	4.37^b^	0.47^a^	21.76
40	0.21^b^	7.79^ab^	7.77^ab^	2.10	2.26^b^	0.00	0.37^ab^	15.77^ab^	4.36^b^	0.37^ab^	20.49
80	0.21^b^	7.51^ab^	7.87^ab^	2.53	2.63^a^	0.00	0.28^ab^	15.59^ab^	5.17^ab^	0.28^ab^	21.03
120	0.23^ab^	6.84^b^	6.90^ab^	2.37	2.64^a^	0.00	0.21^b^	13.98^b^	5.01^ab^	0.21^b^	19.20
160	0.31^a^	6.81^b^	6.68^b^	2.47	2.74^a^	0.00	0.19^b^	13.08^b^	5.21^a^	0.19^b^	19.20

*GNA* gluconapin, *PRO* progoitrin, *NPL* napoleiferin, *GBS* glucobrassicin, *4HGBS* 4-hydroxyglucobrassicin, *SNB* sinalbin, *TROP* glucotropaeolin, *GSLs* glucosinolates.

Means with the same letters do not differ significantly at *p* ≤ 0.05 in Tukey’s test.

In canola-quality *S. alba*, the biosynthesis of sinalbin was inhibited as a result of breeding work. In turn, SAC accumulates more aliphatic and indole GSLs than SAM (the content of progoitrin and napoleiferin is 3 times higher, glucobrassicin content is 8.5 times higher, and 4-hydroxyglucobrassicin content is 6 times higher). Despite the above, the total content of aliphatic and indole GSLs in canola-quality *S. alba* was low (15.2 and 4.8 µM g^–1^ DM, respectively) (Table 5). In SAC, N fertilization induced differences in the qualitative composition of GSLs, but not in the total GSL content of seeds (19.2–21.8 µM g^–1^ DM). Higher N rates decreased the content of progoitrin (120 kg N ha^–1^), napoleiferin (160 kg N ha^–1^), and glucotropaeolin (160 kg N ha^–1^) by 18%, 20%, and 55%, respectively. In turn, N fertilization increased the biosynthesis of gluconapin (160 kg N ha^–1^) and 4-hydroxyglucobrassicin (80 kg N ha^–1^) by 63% and 23%, respectively (Table 5).

#### Tocopherol content

**Fig. 6:**
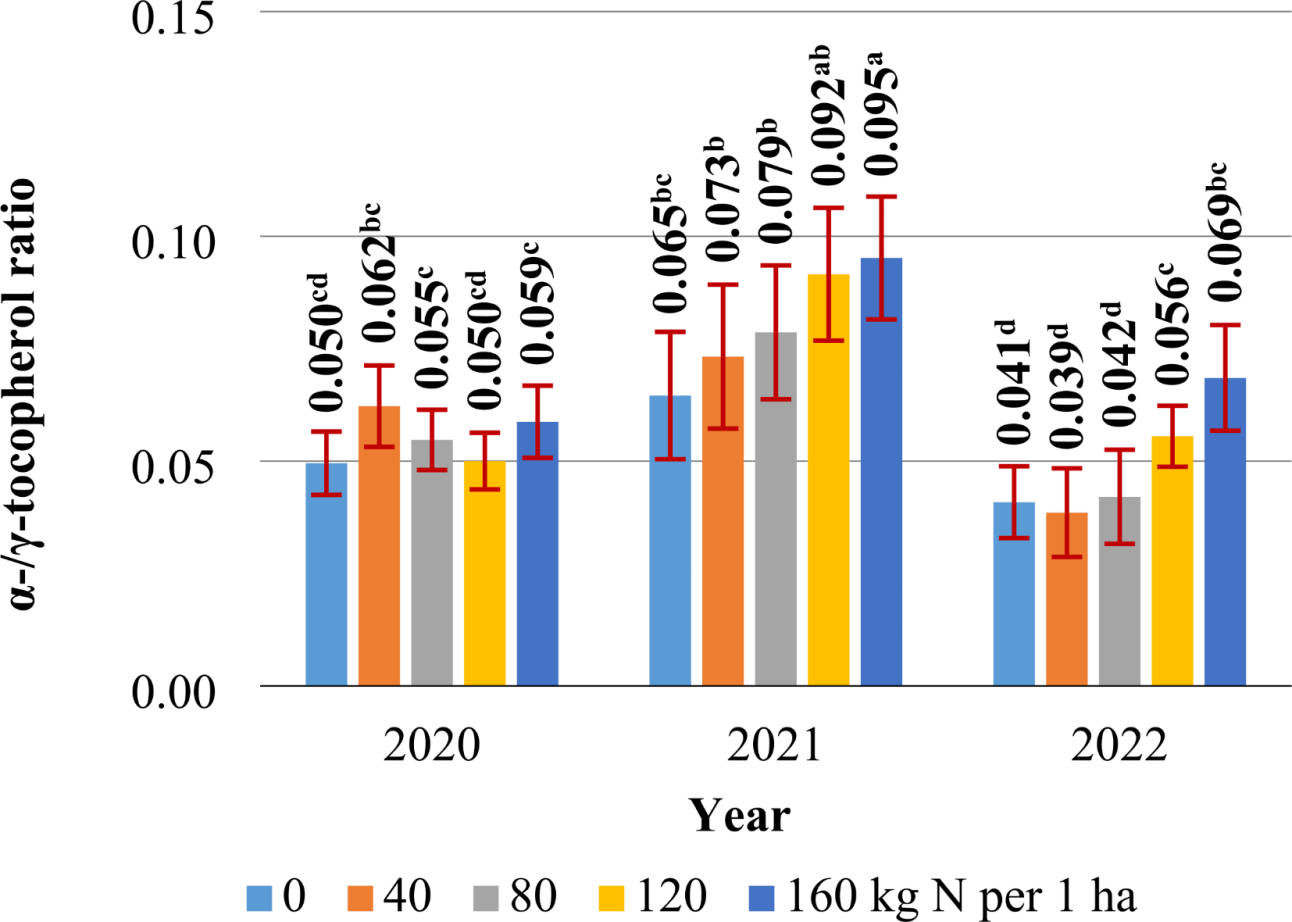
The α-/γ-tocopherol ratio in *S. alba* seeds (2020, 2021, 2022). Means with the same letters do not differ significantly at *p* ≤ 0.05 in Tukey’s test.

The total Ts content of *S. alba* seeds was highest (92.77 mg kg^–1^) in the second growing season (Table 6) which was characterized by the highest total precipitation (45% higher than the long-term average) (Fig. 1). High Ts content resulted from increased biosynthesis of α- and γ- homologs of T. The α-T/γ-T ratio was also highest (0.081) in year 2. The seeds of both white mustard cultivars were most abundant in γ-T (90–94% of total Ts). The content of the remaining T homologs (α-T and δ-T) was very low (4–7% and 3–4% of total Ts, respectively). The content of γ-T and total Ts was 10% and 5% higher, respectively, in SAC seeds. In turn, the content of α-T and δ-T was 48% and 52% higher in SAM seeds. SAC seeds were characterized by a lower α-T/γ-T ratio than SAM seeds (0.047 vs. 0.076). In both cultivars of *S. alba*, the content of α-T, γ-T, and total T increased by 72%, 20%, and 22%, respectively, in response to higher N rates. The α-T/γ-T ratio increased by 42% after the application of 160 kg N ha^–1^ (Table 6). The N rate had no effect on the α-T/γ-T ratio only in the first year of the study (Fig. 6).

**Table 6:** Content of tocopherols in *S. alba* seeds (mg kg^–1^ DM).

Specification	α-tocopherol (mg kg^–1^ DM)	γ-tocopherol (mg kg^–1^ DM)	δ-tocopherol (mg kg^–1^ DM)	Total tocopherols (mg kg^–1^ DM)	α-/γ-tocopherol ratio
Years					
2020	4.22^b^	77.05^b^	2.24	83.51^b^	0.055^b^
2021	6.71^a^	84.06^a^	2.00	92.77^a^	0.081^a^
2022	3.73^b^	75.77^b^	1.94	81.44^b^	0.049^b^
Cultivar, mean for 2020–2022					
Palma (traditional cultivar)	5.84^a^	75.38^b^	2.49^a^	83.71^b^	0.076^a^
Warta (canola-quality cultivar)	3.94^b^	82.54^a^	1.64^b^	88.11^a^	0.047^b^
Nitrogen rate (kg ha^–1^), mean for 2020–2022					
0	3.79^c^	73.19^c^	2.19	79.17^d^	0.052^c^
40	4.32^bc^	74.99^c^	2.10	81.41^cd^	0.058^bc^
80	4.54^bc^	77.64^bc^	2.01	84.19^c^	0.058^bc^
120	5.29^b^	80.95^b^	2.09	88.33^b^	0.066^b^
160	6.51^a^	88.02^a^	1.92	96.45^a^	0.074^a^

Means with the same letters do not differ significantly at *p* ≤ 0.05 in Tukey`s test.

#### Fatty acid composition of oil

**Fig. 7:**
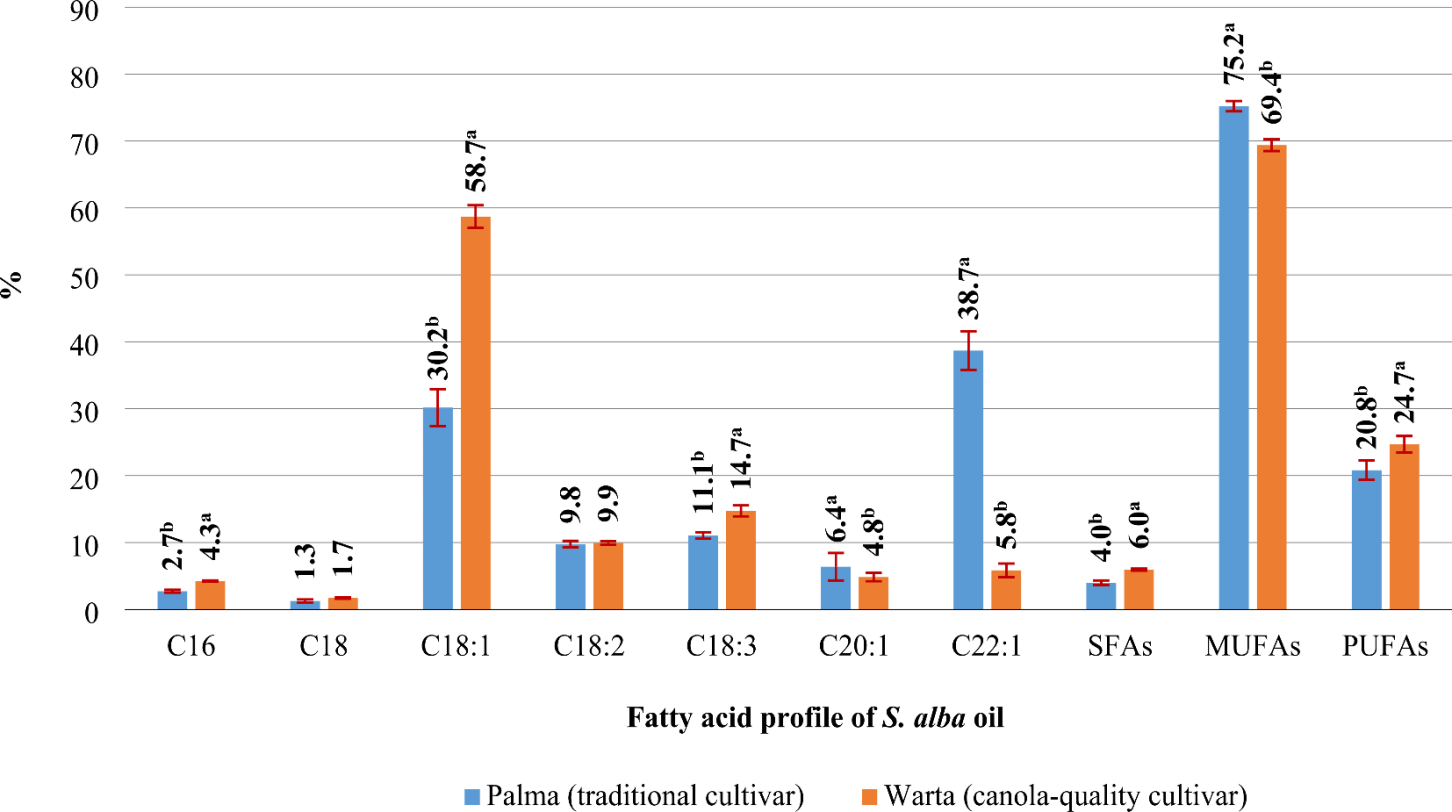
Fatty acid profile of *S. alba* oil (traditional cultivar and canola-quality cultivar), mean for 2020–2022. C16 – palmitic acid; C18 – steric acid; C18:1 – oleic acid; C18:2 – linoleic acid; C18:3 – linolenic acid; C20:1 – eicosanoic acid; C22:1 – erucic acid; SFAs - saturated fatty acids; MUFAs - monounsaturated fatty acids; PUFAs - polyunsaturated fatty acids. Means with the same letters do not differ significantly at *p* ≤ 0.05 in Tukey’s test.

The oil extracted from the seeds of both *S. alba* cultivars was most abundant in monounsaturated fatty acids - MUFAs (69–75%). The share of saturated fatty acids (SFAs) and polyunsaturated fatty acids (PUFAs) in the overall FA profile was determined at 4–6% and 21–25%, respectively. However, the proportion of C22:1, a toxic compound, exceeded 50% of total MUFAs in SAM oil. In SAC oil, the share of C22:1 did not exceed 6%, and the health-promoting C18:1 accounted for nearly 85% of MUFAs (Fig. 7). The rates of N fertilizer had a more pronounced effect on the FA profile of oil in SAC than in SAM (Table 7). In SAM, N fertilization influenced only the content of C18:3 (decrease of 0.8%p in response to 120 kg N ha^–1^). In SAC, N fertilization decreased the content of C18 (40 kg N ha^–1^), C18:1 (80 kg N ha^–1^), and C18:3 (120 kg N ha^–1^), and increased the biosynthesis of C18:2 (80 kg N ha^–1^), C20:1 (80 kg N ha^–1^), and C22:1 (80 kg N ha^–1^) (Table 7).

**Table 7:** Fatty acid profile of *S. alba* oil (%).

Nitrogen rate (kg ha^–1^)	C16	C18	C18:1	C18:2	C18:3	C20:1	C22:1	SFAs	MUFAs	PUFAs
Cv. Palma (traditional cultivar)										
0	2.7	1.0	28.8	9.7	11.6^a^	6.6	39.6	3.8	74.9	21.3
40	2.7	1.2	30.4	9.6	11.2^ab^	6.6	38.3	3.9	75.3	20.8
80	2.7	1.4	30.4	9.7	11.1^ab^	6.1	38.7	4.0	75.2	20.8
120	2.8	1.4	31.2	9.7	10.8^b^	6.3	37.8	4.2	75.3	20.5
160	2.8	1.3	30.0	10.0	10.6^b^	6.3	39.0	4.1	75.3	20.7
Cultivar Warta (canola-quality cultivar)										
0	4.2	1.9^a^	61.5^a^	9.6^b^	15.2^ab^	3.9^b^	3.6^b^	6.2	69.0	24.8
40	4.3	1.6^b^	59.6^ab^	10.0^ab^	15.8^a^	4.3^ab^	4.4^b^	5.9	68.3	25.8
80	4.2	1.7^b^	57.0^b^	10.2^a^	14.7^ab^	5.3^ab^	6.8^a^	5.9	69.1	25.0
120	4.2	1.7^b^	57.5^b^	10.2^a^	14.0^b^	5.2^ab^	7.2^a^	5.9	69.8	24.2
160	4.3	1.6^b^	57.9^b^	9.7^b^	13.9^b^	5.4^a^	7.3^a^	5.9	70.6	23.5

*C16* palmitic acid, *C18* steric acid, *C18:1* oleic acid, *C18:2* linoleic acid, *C18:3* linolenic acid, *C20:1* eicosanoic acid, *C22:1*erucic acid, *SFAs* saturated fatty acids, *MUFAs* monounsaturated fatty acids, *PUFAs* polyunsaturated fatty acids. Means with the same letters do not differ significantly at *p* ≤ 0.05 in Tukey`s test.

## Discussion

### Stand architecture

Stand density affects light access and, indirectly, the development of siliques^74–76^. In the present study, SAC plants were also characterized by shorter stems, but they produced more primary branches than SAM plants^Table 1^. Piętka et al.^56^ also reported that single-low and double-low cultivars of *S. alba* produced shorter stems and flowered earlier than the traditional cultivar.

In the current study, N fertilization influenced plant height and shoot diameter at the base in both cultivars. The greatest increase in plant height was observed in response to 40 kg N ha^–1^. In turn, plants supplied with 80 kg N ha^–1^ produced the thickest shoots^Table 1^. Haq et al.^18^ also found that the height of traditional *S. alba* plants grown in Northern Pakistan continued to increase up to the N rate of 100 kg ha^–1^. In the current study, the N rate was not correlated with the number of primary branches plant^–1^, regardless of cultivar^Table S1^. DuVal et al.^58^ observed that the number of side branches in the traditional cultivar of *S. alba* grown in the Pacific Northwest (Corvallis, OR, USA) increased only after the application of 224 kg N ha^–1^. In the work of DuVal et al.^58^ lower N rates (comparable to those applied in the present study) did not induce significant differences in the number of primary branches plant^–1^ either.

### Yield components

According to research, seed yield is the main qualitative trait in assessments of agronomic practices. The applied production technologies (quantitative and qualitative characteristics of the production process) exert varied effects on seed yield by directly influencing yield-related traits (plants m^–2^, siliques plant^–1^, seeds silique^–1^, and TSW in the production of BOCs)^76–78^. In the present study, seed yields in both cultivars of *S. alba* were determined mainly by the number of plants m^–2^, siliques plant^–1^, and TSW^Table 2^. At harvest, the number of plants m^–2^ was higher in SAM than in SAC. In turn, SAC was characterized by a higher number of siliques plant^–1^, but lower TSW relative to SAM^Table 2^. According to Jankowski et al.^17^, the number of plants m^–2^ and TSW were also higher in SAM. In the cited study, seed yields were significantly influenced by the number of seeds silique^–1^ only in a growing season characterized by a dry spell between sowing and flowering. In this growing season, the number of seeds silique^–1^ was higher in SAM than in SAC^17^. Piętka et al.^56^ also found that TSW was lower in canola-quality *S. alba*. In a study of *B. juncea*, TWS was lower in the canola-quality cultivar than in the traditional cultivar of *B. juncea*^79^.

In spring BOCs cultivated in Poland, N fertilization generally enhances seed yields by increasing the number of seeds plant^–1^ (camelina, oilseed radish) or TSW (white mustard)^17,69^. In the present study, an increase in the N rate to 160 kg N ha^–1^ increased the number of siliques plant^–1^ and decreased TSW^Table 2^. According to Paszkiewicz-Jasińska et al.^78^, these changes can be attributed to the fact that the number of siliques plant^–1^ was positively correlated with the number of seeds silique^–1^ in SAM. However, both parameters were bound by a strong negative correlation with TSW. Therefore, agricultural treatments which increased the number of siliques plant^–1^ and the number of seeds silique^–1^ decreased the weight of *S. alba* seeds^78^. Traditional white mustard responded more favorably to N fertilization in the work of DuVal et al.^58^ and Haq et al.^18^. In the cited studies, N fertilization increased not only the number of siliques plant^–1^ and seeds silique^–1^, but also TSW. In the work of DuVal et al.^58^ and Haq et al.^18^, the beneficial influence of N fertilization on a higher number of seed yield components in *S. alba* could have resulted from a different form of N fertilizer than that applied by Paszkiewicz-Jasińska et al.^78^ and in the current study (urea vs. ammonium nitrate). Nitrogen is released more slowly from urea, and it could have exerted a positive effect on yield components in later stages of growth (by increasing TSW during plant maturation).

### Yield and the harvest index

In spring canola-quality BOCs, seed yields are 10–53% (*B. juncea*) and 10–55% (*S. alba*) lower than in traditional cultivars^17,56,59,79–82,Table 3^. In the present study, canola-quality *S. alba* was characterized by a lower yield potential in all years of the experiment, regardless of weather conditions. Jankowski et al.^17^ reported smaller differences in seed yields between SAM and SAC in growing seasons with high precipitation, but greater differences in dry years. The achievement of comparable yields in canola-quality BOCs requires many years of breeding work^79^ and multifaceted research on agronomic management strategies^17^. In Poland, double-low cultivars of winter oilseed rape achieved comparable seed yields to traditional cultivars only ten years after they had been released for commercial cultivarion^83^. In turn, seed yields in cultivars resistant to clubroot (*Plasmodiophora brassicae* Wor.) approximated the levels reported in traditional cultivars eight years after the first resistant cultivar (Mendel F1) had been registered in Poland^84,85^.

*Brassica* oilseed crops have similar N requirements (50–80 kg N Mg^–1^ seeds)^58,59^. In the current study, both white mustard cultivars also responded similarly to N fertilization. In both SAM and SAC, seed yields increased significantly up to the N rate of 120 kg ha^–1^, regardless of weather conditions in the experimental years^Table 3^. In the wet climate of the Pacific Northwest, seed yields in the traditional white mustard cultivar increased up to the N rate of 168–224 kg ha^–158^. The responses of new BOC cultivars (where the chemical composition of seeds and the metabolism of bioactive compounds have been modified) to N fertilization remains insufficiently investigated^17^. In Canada, the N rate of 100 kg ha^–1^ induced a greater increase in seed yields in the canola-quality cultivar than the traditional cultivar of *B. juncea*^60^. In the work of Jankowski et al.^17^, a similar increase was observed in the seed yields of *S. alba* cultivars (canola-quality and traditional) after the application of 80 (dry year) and 120 kg N ha^–1^ (wet year). The varied responses of *S. alba* to N fertilization can be attributed to the fact that the optimal N rate is determined by the yield potential of a given species/cultivar, as well as weather conditions that affect the mineralization and uptake of soil N^86^.

Despite a high demand for N, BOCs are generally characterized by low NFUE. In comparison, the NFUE of common wheat has been determined at 33–47 kg grain kg^–1^ N on average^87,88^. In the present study, the NFUE of *S. alba* reached 16.1 (SAC) and 19.1 kg seeds kg^–1^ N (SAM)^Table 3^. Similar values were reported by Gan et al.^60^ in Canada. In the cited study, *S. alba* was characterized by the lowest NFUE in the group of spring BOCs (*B. rapa*, *B. napus*, *B. juncea*). In the present study, the NFUE peaked in both white mustard cultivars after the application of 40 kg N ha^–1^. The analyzed parameter decreased in response to the optimal N rate (120 kg ha^–1^)^Table 3^. Higher N rates also decreased the NFUE in other BOCs (camelina, Indian mustard, agrimony, oilseed radish, spring rapeseed, and winter rapeseed)^60,66,68,69,89^.

In the current study, the HI of *S. alba* was determined at 0.44–0.50. In north-eastern Poland, similar values of HI were reported in spring camelina^69^, whereas much lower values were noted in oilseed radish^66^. In the present study, no significant differences were observed in the HI of the analyzed *S. alba* cultivars^Table S1^. Similar values of HI were reported by Gan et al.^60,81^ in canola-quality and traditional cultivars of *B. juncea*. In turn, Malhi et al.^80^ found that the HI was 30–33% higher in traditional than canola-quality *B. juncea*. According to the cited authors, the lower HI of canola-quality *B. juncea* resulted from a greater reduction in seed yield due to water deficit during plant maturation, compared with the traditional cultivar. In the current experiment, precipitation levels during *S. alba* maturation approximated the long-term average in all years. In turn, insufficient rainfall during the early stages of plant growth, observed in the first year of the study, caused a greater decrease in the straw yield of SAM than SAC. Therefore, in the present study, the HI was higher in SAC than in SAM only in the year with the lowest total precipitation^Fig. 4^. A relationship between precipitation and HI values in different BOC cultivars (canola-quality vs. traditional) was also observed by Mustafa et al.^90^. In this study, N fertilization was not correlated with the HI of traditional and canola-quality cultivars of *S. alba*^Table 1^. Nitrogen did not influence the HI of traditional white mustard in the work of DuVal et al.^58^, either. In turn, Haq et al.^18^ reported that the HI of traditional white mustard increased after the application of 80 kg N ha^–1^. A further increase in the N rate led to a decrease in the HI. In the study by Haq et al.^18^, N rates higher than 80 kg ha^–1^ induced a greater decrease in seed yields than straw yields. In turn, in the work of DuVal et al.^58^ and in the present study^Table 3^, even high N rates did not lead to a significant decrease in seed yields or straw yields. According to Grzebisz^86^, excess N can promote vegetative growth at the expense of seed production, which can result in a proportionally greater decrease in seed yields than straw yields (lower HI). Under exposure to high N rates, plants produce more vegetative biomass, which can decrease photosynthetic efficiency by inhibiting light access to lower leaves and side branches. As a result, less energy is available for seed production, which decreases seed yields and the HI^86^.

### Seed and oil quality

#### Fat, protein, and fiber content of seeds

In the present study, the seeds of canola-quality *S. alba* had a higher content of crude fat and crude fiber, and a similar content of total protein, compared with the traditional cultivar^Table 4^. In a study by Piętka et al.^56^, SAC seeds accumulated more crude fat and less total protein than SAM seeds. Crude fat content was higher in the seeds of a white mustard cultivar with a low content of C22:1 in a study by Sawicka et al.^91^. Beckie et al.^82^ also found that crude fat content was higher in canola-quality *B. juncea* than in the traditional cultivar of *B. juncea*. Similarly to the present study, no differences were reported in the protein content of seeds in the compared *B. juncea* cultivars. Malhi et al.^80^ found no differences in the total protein content of seeds in canola-quality and traditional *B. juncea*.

In BOCs, N fertilization generally decreases crude fat content, but increases the total protein content of seeds. These correlations were validated in numerous studies of winter oilseed rape and spring BOCs, including camelina, white mustard, Indian mustard, and oilseed radish^18,66,69,92,93,Table 4^. In the current study, the N rates of 80 and 160 kg ha^–1^ decreased crude fat content and increased the total protein content of seeds in both cultivars of *S. alba*. In addition, the N rate of 120 kg ha^–1^ increased the crude fiber content of seeds in both SAC and SAM^Table 4^. In other studies, N fertilization also led to a considerable increase in the crude fiber content of camelina^69^ and oilseed radish^66^.

#### Glucosinolate content of seeds

Glucosinolates are biologically active compounds that are commonly found in plants of the family *Brassicaceae*^36,94–96^. In *Brassica* species and their cultivars, GSL concentrations differ in various plant organs^36,95^. The content of GSLs in the roots and straw of traditional *S. alba* was determined at 0.18–0.97 and 2.27–5.67 µM g^–1^ DM, respectively^36,97^. It should also be noted that white mustard is the only species of spring BOCs (crambe, Indian mustard, camelina, rapeseed, oilseed radish) to accumulate more GSLs in roots than in straw^36,97^. The content of GSLs is highest in seeds of traditional *S. alba* (93–308 µM g^–1^ DM)^36,38,56^. Sinalbin (indole GSL) is the predominant GSL in traditional white mustard, and its content reaches 52–78% of total GSLs in roots, 78–99% in straw^36,97^, and 96–98% in seeds^36,37,Table 5^. In this experiment, the content of GSLs was 8–10 lower in the seeds of canola-quality than traditional white mustard^Table 5^. The seeds of canola-quality *S. alba* were also much less abundant in GSLs in the work of Piętka et al.^56^. The present study demonstrated that aliphatic GSLs are predominant in the seeds of canola-quality *S. alba.* The content of aliphatic GSLs in seeds was higher in SAC than SAM^Table 5^, but it did not exceed the maximum value for canola-quality *S. alba* in the EU (25 µM g^–1^ 91% DM seeds)^98^. Similar variations in the quantitative and qualitative composition of GSLs in the seeds of canola-quality and traditional white mustard were reported by Piętka et al.^56^.

In BOCs, GSL synthesis is determined mainly by genetic and ecophysiological traits, as well as some agricultural treatments, in particular fertilization^36,95,99–101^. In the present study, the content of indole GSLs increased in SAC seeds in response to N fertilization^Table 5^, which is a desirable outcome because the decomposition products of this GSL fraction have anti-carcinogenic properties^95^. In the work of Zhao et al.^102^ increased N supply also decreased the biosynthesis of aliphatic GSLs and promoted the accumulation of indole GSLs, thus increasing the suitability of fat-free seed residues for feed production. In the current study, N fertilization decreased total GSL content in the cultivar characterized by high GSL concentrations (SAM) by decreasing the proportions of aliphatic GSLs (progoitrin and napoleiferin) and aromatic GSLs (sinalbin). In the cultivar with low GSL concentration (SAC), N fertilization did not induce significant differences in the total GSL content of seeds, but it strongly influenced the qualitative composition of GSLs (by increasing the content of gluconapin and 4-hydroxyglucobrassicin, and decreasing the content of progoitrin, napoleiferin, and glucotropaeolin)^Table 5^.

#### Tocopherol content of seeds

The seeds of BOCs are a rich source of Ts which are natural antioxidants^33,103–105^ that play an important role in the oxidative stability of vegetable oils^103,104^. The oil extracted from the seeds of *Brassicaceae* family plants is most abundant in γ-T. The highest concentration of γ-T is found in camelina, oilseed radish, and white mustard oil (706–769 mg kg^–1^, i.e. 91–96% of total Ts). Safflower (*Carthamus tinctorius* L.), sunflower (*Helianthus annuus* L.), milk thistle [*Silybum marianum* (L.), Gaertner], and niger seed [*Guizotia abyssinica* (L.f.) Cass] oil is abundant in α-T (612–686 mg kg^–1^, i.e. 91–95% of total Ts). The oil derived from amaranthus (*Amaranthus cruentus* L.) seeds is characterized by a high content of β-T (334 mg kg^–1^, i.e. 42% of total Ts). *Brassica* oilseed crops are nearly devoid of β-T. In turn, δ-T is the predominant T homolog in starflower seeds (*Borago officinalis* L.) (1172 mg kg^–1^, i.e. 91% of total Ts)^33^. The T content of white mustard seeds ranges from 81 to 93 to 154–198 mg kg^–1^ seeds^106,Table 6^ and 842–952 mg kg^–1^ oil^29,33^. Tocopherol levels in white mustard are similar to those noted in Ethiopian mustard^107,108^, but much higher than those reported in Indian mustard (by 32–38%) and black mustard (by 49–52%)^109^.

In the present study, SAC seeds contained more γ-T and total Ts than SAM seeds. In turn, SAM seeds were more abundant in α-T and -T. -T was not identified in *S. alba* seeds ^Table 6^. The oil extracted from *S. alba* cultivars was also free of -T in the work of Gawrysiak-Witulska et al.^29^. In the present study, the seeds of canola-quality white mustard were characterized by a lower α-T/γ-T ratio than traditional white mustard seeds^Table 6^. Oils with a low α-T/-T ratio, in particular oils containing FAs with a low degree of unsaturation, are characterized by higher thermal stability and are more suitable for high heat-heat cooking^110–112^. In turn, oils with a high α-T/-T ratio are better suited for low-heat cooking because α-T is characterized by the highest vitamin E activity^113^. It should be noted that the T content of BOCs is also determined by genotype^114–116^. Egesel et al.^114^ found that T levels were higher in open-pollinated than hybrid cultivars of rapeseed. Verma et al.^116^ reported that the T content of *B. juncea* was bound by a negative correlation with C22:1 and GSL concentrations. Cultivars with low levels of C22:1 and GSLs contained more Ts than traditional cultivars. In turn, Gupta et al.^115^ found that an Indian genotype of *B. juncea* (high in GSLs) contained more Ts than the East European genotype (low in GSLs). In the work of Gawrysiak-Witulska et al.^29^, the content of Ts in the oil extracted from the double-low cultivar of *S. alba* was comparable with that noted in the oil of a single-low cultivar with reduced concentration of C22:1. The oil extracted from the single-low cultivar was only somewhat more abundant in -T than the oil derived from the double-low cultivar. The content of α-T and -T in seeds was similar in both cultivars^29^.

In the current study, a higher supply of N increased α-T and γ-T levels, the α-T/γ-T ratio, and the content of total Ts in both *S. alba* cultivars^Table 6^. According to Egesel et al.^113^ and Hussain et al.^117^, Ts biosynthesis in BOCs is influenced by the N management strategy, where insufficient N levels inhibit the biosynthesis and accumulation of Ts in seeds. In the work of Egesel et al.^114^, an N rate of 130 kg ha^–1^ significantly increased the total Ts content of rapeseed, mainly by increasing the concentration of γ-T. Higher N rates decreased total Ts content, mainly by inhibiting the biosynthesis of α-T. Szatkowski et al.^66^ also found that the T content of oilseed radish seeds decreased in response to N rates higher than 90 kg ha^–1^ (α- and γ-T levels decreased). Nitrogen rates below 90 kg ha^–1^ induced a 28% increase in the total Ts content of oilseed radish seeds by increasing α-, β-, and γ-T levels. In contrast, Hussain et al.^117^ reported that the T content of rapeseed increased only after the application of a very high N rate (270 kg ha^–1^).

#### Fatty acid composition of oil

The first breeding efforts aiming to modify the composition of oil derived from BOCs were undertaken by Stevenson in the 1960s, and this goal was accomplished by replacing C22:1 with C18:1^50^. The oil extracted from canola-type white mustard contains around 59–68% of C18:1, 11–15% of C18:3, 10–14% of C18:2, 4% C20:1, and 6% of C22:1^57,Fig. 77^. In this experiment, SAC oil was more abundant in C16, C18:1, and C18:3 than SAM oil. In turn, SAM oil was a richer source of C20:1 and C22:1 (long-chain MUFAs) than SAC oil^Fig. 7^.

In the group of agronomic factors, the FA composition of oil is affected mostly by the harvest strategy and mineral (mainly N) fertilization^118–120^. In the present study, N fertilization exerted different effects on the FA profile of oil extracted from the analyzed *S. alba* cultivars. In the traditional white mustard cultivar, the N rate of 120 kg ha^–1^ affected only the content of C18:3. In turn, N fertilization in SAC decreased the content of C18, C18:1, and C18:3, but enhanced the biosynthesis of C18:2, C20:1, and C22:1^Table 7^. The FA profile of BOC oil is a genetic/varietal trait that is modified by environmental conditions and agronomic practices^121,122^. For this reason, N fertilization does not induce unidirectional (repeatable) changes in the FA profile of BOC oil. In the work of Jankowski et al.^69^, N fertilization increased the share of C18:2 and C18:3, and decreased the proportions of C18:1 and C20:1 in camelina. In a study by Szatkowski et al.^66^, the application of 30 kg N ha^–1^ decreased the content of C22:1 and increased the concentrations of C18:3 and C20:1 in oilseed radish oil. A further increase in the N rate (90 kg ha^–1^) increased the content of SFAs (C16 and C18).

## Conclusions

The study demonstrated that in temperate climates (north-eastern Poland), the highest seed and straw yields of both *S. alba* cultivars are achieved in response to an N rate of 120 kg N ha^–1^. The seeds of the analyzed *S. alba* cultivars have different industrial applications, which is why their processing suitability is determined by different quality parameters. The seeds of canola-quality *S. alba* are a valuable resource in the production of edible oil and animal feed, which is why they should be characterized by a high content of crude fat, total protein, and Ts, a low content of aliphatic and aromatic GSLs, and a high content of indole GSLs. These parameters were optimized after the application of 120–160 kg N ha^–1^. It should also be noted than a further increase in the N rate to 160 kg N ha^–1^ did not decrease SAC yields and improved the processing suitability of seeds. In turn, SAM seeds are used in the production of mustard and oleochemicals, and they should be characterized by a high content of crude fat, total protein, aromatic GSLs, and C22:1. The above parameters improved when SAM was supplied with 80–120 kg N ha^–1^. Nitrogen rates higher than 120 kg N ha^–1^ induced a significant decrease in the content of crude fat and aromatic and total GSLs in seeds.

The results of this study indicate that the yield and quality of *S. alba* seeds are determined by genotype and the N rate. The study confirmed that the seeds and oil of canola-quality *S. alba* have a high nutritional value, but satisfactory seed yields were not achieved. Therefore, breeding efforts should focus on improving yields in selected genotypes of canola-quality *S. alba*. Fertilizer management strategies for different climate zones should be optimized to increase yields and seed quality in *S. alba*, and to reduce the carbon footprint. Further research is also needed to enhance the nutritional value and the content of bioactive compounds in *S. alba* seeds and oil used in the production of special-purpose foods, pharmaceuticals (functional foods, nutraceuticals), and cosmetics.

## Electronic supplementary material

Below is the link to the electronic supplementary material.

Supplementary Material 1

## Data Availability

All data generated or analysed during this study are included in this published article.
