# Supplementary material for: The effect of nitrogen management on seed yield and quality in traditional and canola-quality white mustard
Source: Sci Rep. 2024 Oct 30;14:26127. doi: 10.1038/s41598-024-76582-9 (PMC11525544; doi:10.1038/s41598-024-76582-9)
Supplement: Supplementary file 1 — Supplementary Material 1 [file 41598_2024_76582_MOESM1_ESM.docx]

**Table S1**

Split-split-plot ANOVA and *F*-test statistics (3 significant figures).

| Variable | Y *F*_2/4_ | Cv. *F_1/_*_6_ | Y × Cv. *F_2/_*_6_ | N *F_4/_*_48_ and *F_3/_*_36 (NFUE)_ | Y × N *F_8/_*_48_ and *F_6/_*_36 (NFUE)_ | Cv. × N *F_4/_*_48_ and *F_3/_*_36 (NFUE)_ | Y × Cv. × N *F_8/_*_48_ and  *F_6/_*_36 (NFUE)_ |
| --- | --- | --- | --- | --- | --- | --- | --- |
| Plant height (cm) | 7.38* | 16.1** | 0.85*ns* | 8.85*** | 2.49* | 0.74*ns* | 0.82*ns* |
| Shoot diameter at the base (mm) | 52.9*** | 0.20*ns* | 4.32*ns* | 6.32*** | 2.15* | 2.71* | 1.12*ns* |
| Primary branches plant^–1^ | 118*** | 10.4* | 2.64*ns* | 1.88*ns* | 0.44*ns* | 0.89*ns* | 1.16*ns* |
| Plants m^–2^ | 0.06*ns* | 19.4** | 0.68*ns* | 1.80*ns* | 0.88*ns* | 0.28*ns* | 1.25*ns* |
| Siliques plant^–1^ | 0.12*ns* | 19.5** | 0.95*ns* | 9.78*** | 0.57*ns* | 0.82*ns* | 1.04*ns* |
| Seeds silique^–1^ | 74.2*** | 1.09*ns* | 10.5* | 1.05*ns* | 0.96*ns* | 0.13*ns* | 0.84*ns* |
| 1000-seed weight (g) | 128*** | 953*** | 14.3** | 5.28** | 0.86*ns* | 2.79* | 0.90*ns* |
| Seed yield (Mg ha^–1^) | 0.48*ns* | 22.9** | 0.62*ns* | 31.2*** | 0.68*ns* | 2.49*ns* | 0.56*ns* |
| NFUE (kg seed per 1 kg N) | 0.71*ns* | 10.0* | 0.97*ns* | 385*** | 0.64*ns* | 0.41*ns* | 0.93*ns* |
| Straw yield (Mg ha^–1^) | 9.47*** | 22.7** | 5.78* | 7.74*** | 5.14*** | 0.46*n*s | 1.33*ns* |
| Harvest index | 7.26* | 2.02*ns* | 12.7** | 1.95*ns* | 2.71* | 1.98*ns* | 1.45*ns* |
| Crude fat content of seeds (g kg^–1^ DM) | 5.31*ns* | 25.7** | 14.4** | 10.7*** | 0.65*ns* | 0.87*ns* | 0.54*ns* |
| Total protein content of seeds (g kg^–1^ DM) | 5.14*ns* | 157*** | 12.8** | 22.9*** | 0.80*ns* | 4.66** | 3.39** |
| Crude fiber content of seeds (g kg^–1^ DM) | 27.4** | 31.5** | 14.3** | 10.5*** | 1.77*ns* | 1.39*ns* | 0.94*ns* |
| Acid detergent fiber (%) | 7.33* | 3.19*ns* | 2.93*ns* | 7.03*** | 0.25*ns* | 2.65* | 0.52*ns* |
| Neutral detergent fiber (%) | 94.0*** | 7.06* | 0.89*ns* | 1.00*ns* | 1.81*ns* | 0.97*ns* | 2.13*ns* |
| α-tocopherol in seeds  (mg kg^–1^) | 59.5** | 97.9*** | 9.89* | 17.7*** | 2.01*ns* | 0.770*ns* | 0.409*ns* |
| γ-tocopherol in seeds (mg kg^–1^) | 136*** | 87.9*** | 1.19*ns* | 39.6*** | 2.02*ns* | 2.38*ns* | 0.482 *ns* |
| δ-tocopherol in seeds (mg kg^–1^) | 0.568*ns* | 131*** | 2.41*ns* | 0.579*ns* | 0.279*ns* | 2.49*ns* | 0.496*ns* |
| Σ tocopherols in seeds (mg kg^–1^) | 227*** | 23.6** | 0.08*ns* | 44.8*** | 1.66*ns* | 2.13*ns* | 0.472*ns* |
| α-/γ-tocopherol | 41.8** | 165*** | 12.5** | 7.46*** | 2.22* | 0.512*ns* | 0.253*ns* |

* significant at *P*≤0.05; **significant at *P*≤0.01; *** significant at *P*≤0.001; *ns* – not significant. Y- growing season; Cv. – cultivar; N – nitrogen rate; *F*_numerator_ *_k_*_-1 degrees of freedom/denominator error degrees of freedom;_ NFUE - nitrogen fertilizer use efficiency.

**Table S2**

*F*-test statistics for randomized block design ANOVA in separate analyses of white mustard cultivars Palma and Warta for the glucosinolate content of seeds and the fatty acid profile of oil (3 significant figures).

| Variable | cv. Palma *F*_4/38_ | cv. Warta *F*_4/38_ |
| --- | --- | --- |
| Gluconapin (μM g^–1^ DM seeds) | - | 3.62* |
| Progoitrin (μM g^–1^ DM seeds) | 5.17** | 2.01*ns* |
| Napoleiferin (μM g^–1^ DM seeds) | 4.27** | 2.49*ns* |
| Glucobrassicin (μM g^–1^ DM seeds) | 1.07*ns* | 1.38*ns* |
| 4-hydroxyglucobrassicin (μM g^–1^ DM seeds) | 3.25* | 10.7*** |
| Sinalbin (μM g^–1^ DM seeds) | 10.6*** | - |
| Glucotropaeolin (μM g^–1^ DM seeds) | - | 14.8*** |
| Aliphatic glucosinolates (μM g^–1^ DM seeds) | 3.95** | 3.37*** |
| Indole glucosinolates (μM g^–1^ DM seeds) | 1.61*ns* | 3.67* |
| Aromatic glucosinolates (μM g^–1^ DM seeds) | 9.88*** | 7.29*** |
| Total glucosinolates (μM g^–1^ DM seeds) | 9.62*** | 1.33*ns* |
| Palmitic acid (%) | 0.12*ns* | 0.29*ns* |
| Steric acid (%) | 1.00*ns* | 5.14** |
| Oleic acid (%) | 0.20*ns* | 3.44* |
| Linoleic acid (%) | 0.21*ns* | 2.93* |
| Linolenic acid (%) | 1.34*ns* | 2.70* |
| Eicosanoic acid (%) | 0.017*ns* | 3.12* |
| Erucic acid (%) | 0.10*ns* | 15.2*** |
| Saturated fatty acids (%) | 0.48*ns* | 1.42*ns* |
| Monounsaturated fatty acid (%) | 0.02*ns* | 1.31*ns* |
| Polyunsaturated fatty acid (%) | 0.33*ns* | 2.73* |

* significant at *p* ≤ 0.05; **significant at *p* ≤ 0.01; *** significant at *p* ≤ 0.001; *ns* – not significant; *F*_numerator_ *_k_*_-1 degrees of freedom/denominator error degrees of freedom_.
